# Case Report: Sequential fetal reduction in a triplet pregnancy and its effects on coagulation balance

**DOI:** 10.3389/fgwh.2026.1786320

**Published:** 2026-04-29

**Authors:** Erzsebet Nagy, Ira Bernstein, Maria Cristina Bravo

**Affiliations:** 1 University of Vermont Larner College of Medicine, Burlington, VT, United States; 2 The Department of Obstetrics, Gynecology, and Reproductive Sciences, University of Vermont Larner College of Medicine, Burlington, VT, United States; 3 The Department of Pathology and Laboratory Medicine, University of Vermont Larner College of Medicine, Colchester, VT, United States

**Keywords:** assisted reproduction (ART), coagulation, fetal reduction, thromboelastography, high-risk pregnancy, triplet pregnancy

## Abstract

Over the last few decades, the increase in medically assisted reproduction has led to a significant increase in the frequency of twin and higher-order multiple pregnancies (defined as triplets or more). A critical element of this trend is the increased risk of preterm birth and infant mortality in twin and higher-order pregnancies. Fetal reduction (FR) was initially established as an option to reduce these adverse outcomes. Indications for FR have now expanded, with literature indicating clear improvement of outcomes after reduction to twins or singletons from triplet pregnancies, although physiological adaptations during pregnancy following FR have not been thoroughly investigated. One clinically relevant research gap is the effect of FR on coagulation and fibrinolysis, given that pregnancy is associated with a hypercoagulable state. In this novel case study, our objective was to characterize the hemostatic impact of FR. We employed a functional assay, thromboelastography (TEG), to compare the maternal hemostatic function in a triplet pregnancy that was sequentially reduced to a singleton pregnancy with uncomplicated singleton pregnancies. Patient 611 became pregnant during her fourth cycle of intrauterine insemination, and a dating ultrasound confirmed a trichorionic triamniotic triplet pregnancy. After counseling, the patient chose to undergo fetal reduction to a singleton pregnancy. Both reductions were achieved via the injection of potassium chloride. TEG was performed on blood samples taken during pregnancy, and patient 611 was compared with patients with singleton pregnancies who had also undergone ovulation induction at similar gestational ages. We observed an overall decrease in coagulability across all TEG parameters from prepregnancy to term in patient 611 compared with that in control singleton pregnancies. These data suggest that fetal reduction may limit the pregnancy-associated hypercoagulable states.

## 1 Introduction

Over the last few decades, the increase in medically assisted reproduction (MAR) has led to an increase in the number of twin and higher-order multiple pregnancies (defined as triplets or more). Between 1980 and 2009, a period often referred to as “an epidemic of multiple pregnancies,” the rate of twin births increased by 76% (1). The rate of multiple pregnancies peaked in the late 1990s and has since subsided with improved guidelines for MAR procedures. Nevertheless, despite the updated guidance, the occurrence of twins remains significantly higher than expected with spontaneous conception (2–5). The rise in multiple pregnancies, coupled with the well-documented increased risk of preterm birth and infant mortality in twins and higher-order pregnancies, has resulted in a national challenge (6, 7).

To reduce the adverse outcomes associated with multiple pregnancies, fetal reduction (FR) was established in the 1980s to mitigate the increased perinatal risk. The procedure involves the selective termination of one or more fetuses. The indications for FR have expanded, with the literature indicating a clear improvement in outcomes after reduction to twins or singletons from triplet or higher-order pregnancies (5). Most of the literature on FR focuses on maternal and fetal outcomes, although specific physiological changes in pregnant individuals after FR haven't been thoroughly investigated. However, the effects of FR on coagulation and fibrinolysis remain unknown. As pregnancy is well established as a hypercoagulable state with an increased risk of thrombotic events, there are theoretical concerns that could link placental devascularization with this prothrombotic risk (8). There is evidence of differences in coagulation-fibrinolysis characteristics between singleton and twin pregnancies, although longitudinal changes across gestation in twin and higher-order pregnancies are unclear, and the influence of FR on coagulation balance is unknown (9, 10).

In this case study, we examined hemostatic function using the functional assay thromboelastography (TEG) across the gestational period of a triplet pregnancy that was sequentially reduced to a singleton pregnancy. The findings are compared to established singleton pregnancies where ovulation induction was also employed.

## 2 Case description

Patient 611 was a 33-year-old G1P0 female who enrolled in our IRB-approved protocol for people using pharmacologic stimulation and intrauterine insemination (IUI) to achieve pregnancy. She had no known coagulopathies or chronic medical diseases, a non-contributory family history, and an unremarkable social history. She became pregnant during her fourth cycle of IUI. A dating ultrasound at 9 weeks confirmed a trichorionic triamniotic triplet pregnancy. First-trimester ultrasonography revealed that each fetus exhibited normal nuchal translucency. Physical examinations and laboratory values across the pregnancy were unremarkable until she reached 40w6d, at which point she met the criteria for preeclampsia without severe features based on two mild range blood pressure elevations and a urine protein/ creatinine ratio of 0.82.

## 3 Timeline

See Figure 1.

**Figure 1:**
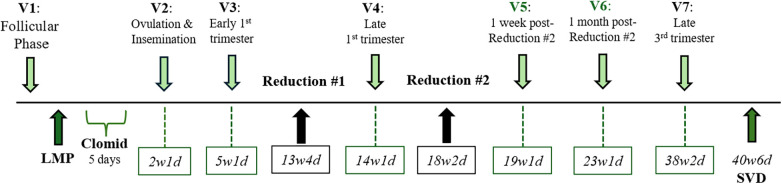
Timeline of study visits, interventions, and assessments during pregnancy. Visit 1 took place during the follicular phase for all subjects. After subjects' last menstrual period (LMP), they began ovulation induction with five days of Clomid. All subjects participated in visits 2 and 3, and for those that had a stable pregnancy, visits 4 and 7 during which blood samples were collected. Boxes that correspond to visits indicate gestational age in weeks and days of patient 611 at these visits. Visits 5 and 6 are specific to patient 611 to more closely assess changes in coagulation parameters after her reductions. Patient 611 had a spontaneous vaginal delivery (SVD) at 40w6d.

## 4 Diagnostic assessment

This study examined the longitudinal changes in coagulation balance over the course of a triplet pregnancy that was sequentially reduced to a singleton pregnancy. Whole blood samples were collected at visits, as outlined in the timeline in Figure 1. TEG was performed to determine the clot formation time (R), clot strength (MA), and fibrinolysis (LY30). For comparison, TEG data from 15 other participants enrolled in our study who underwent ovulation induction (V1–V3), five of whom conceived singleton pregnancies (V4 and V7), are presented. This control group was used because these pregnancies were also achieved with ovulation induction and IUI, and blood samples were collected at comparable gestational windows.

An ultrasound conducted at 9 weeks of gestation confirmed the presence of a trichorionic triamniotic triplet pregnancy, and a subsequent first-trimester ultrasound revealed that each fetus exhibited normal nuchal translucency. Because the patient and her partner were concerned about the risk of complications with triplets, they chose to pursue fetal reduction to a singleton pregnancy. The first fetal reduction of a triplet to twin pregnancy occurred at 13 weeks gestation via potassium chloride injection, and the third fetus was selected for reduction. During the same procedure, chorionic villus sampling (CVS) was performed on the second fetus due to the placental position ( CVS could not be performed on the first fetus because of its posterior placental location; consequently, through shared-decision making, the patient chose to undergo amniocentesis for the first fetus at a later gestational age). The patient experienced rupture of the amniotic sac of the third fetus postprocedure, but she recovered well. The CVS results for the second fetus were normal. At 16 weeks gestation, the patient underwent amniocentesis for the first fetus. These results were also normal; therefore, the second reduction was based on the position of the remaining twins. The second reduction to a singleton pregnancy occurred at 18w1d and was also performed using a potassium chloride injection. In addition, she underwent labor induction at 40w6d because she met the criteria for preeclampsia without severe features. She gave birth at 41w0d via spontaneous vaginal delivery to a healthy, vigorous infant weighing 3,280 g with Apgar scores of 9 and 9.

Serial TEG measurements were obtained from prepregnancy through term for patient 611 (reported values of duplicates are presented as mean ± standard deviation) and compared with the TEG values from singleton pregnancies conceived via ovulation induction.

### 4.1 Clotting time (R)

**Figure 2:**
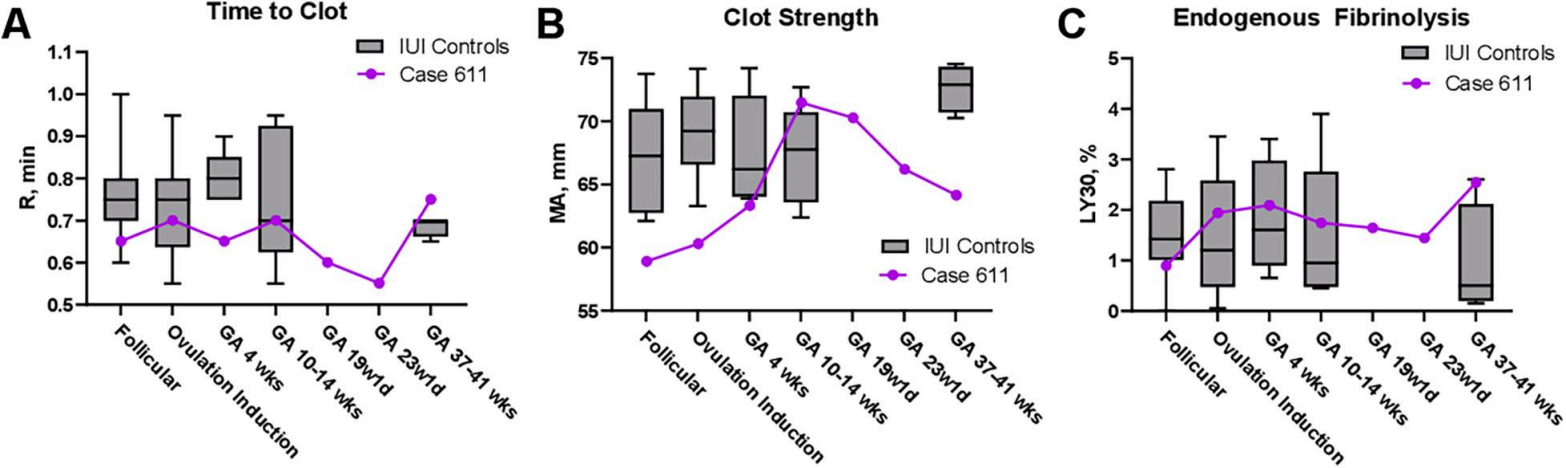
Assessment of coagulation parameters in patient 611 compared to intrauterine insemination (IUI) controls. Coagulation parameters were measured using thromboelastography (TEG) at multiple time points throughout pregnancy in patient 611 (purple line) and IUI controls (gray box and whisker plots). **(A)** Time to clot (R, min) shows clot formation time, where lower values indicate a more hypercoagulable state. **(B)** Clot strength (MA, mm) correlates with platelet function, where higher values indicate stronger clotting and more robust platelet function. **(C)** Endogenous fibrinolysis (LY30 %) indicates the amount of clot breakdown at 30 min, where lower values indicate a resistance to fibrinolysis.

The time to clot was determined using the parameter R, where lower values indicate a hypercoagulable state (Figure 2A). Throughout the course of 611's pregnancy, the time to clot mildly increased from prepregnancy (0.65 ± 0.07 min) to term (0.75 ± 0.07 min), indicating a slight decrease in coagulability. Control patients exhibited mild decreases or stability in the time to clot across pregnancy, indicating a slight increase in coagulability.

### 4.2 Clot strength (MA)

Clot strength, as reported by MA (maximum amplitude), is highly associated with platelet function (Figure 2B). Throughout the course of 611's pregnancy, clot strength increased steeply from prepregnancy (59.0 ± 2.1 mm) to shortly after the initial reduction when it peaked (71.6 ± 0.2 mm). Subsequently, clot strength exhibited a downward trend by the end of the term (64.2 ± 1.8 mm). This is different from the pattern identified in control singleton pregnancies, as these patients exhibited more stability in clot strength throughout the first trimester of the pregnancy (67.1 – 69.1 mm) until term, when there was an increase (72.7 ± 1.9 mm).

### 4.3 Fibrinolysis (LY30)

Endogenous fibrinolysis was determined using LY30, where lower values indicate increased resistance to fibrinolysis (Figure 2C). Throughout the course of 611's pregnancy, fibrinolysis fluctuated but ultimately increased from prepregnancy (0.90 ± 0.28 %) to the end of term (2.55 ± 0.92 %), indicating a decreasing resistance to fibrinolysis. This was different from the IUI control patients, who had a decrease in fibrinolysis (and thus an increase in coagulability) from prepregnancy to the term.

## 5 Discussion

This novel case study is the first to examine longitudinal changes in coagulation balance over the course of a triplet pregnancy that was sequentially reduced to a singleton pregnancy. Although pregnancy is known to be a hypercoagulable state with an increased risk of thrombotic events, and multiple gestations are associated with increased perinatal risk, the hemostatic effect of FR has not been previously characterized. By comparing this patient's TEG trajectory with those of uncomplicated singleton pregnancies conceived via ovulation induction and IUI, this case is the first to provide insight into potential alterations in coagulation dynamics following FR.

Throughout pregnancy, patient 611 demonstrated a mild increase in the clotting time (R), an increase and subsequent decrease in platelet function (MA), and a net increase in fibrinolysis (LY30). These patterns diverge from the progressive hypercoagulability observed in our control group, which generally showed a stable or shortened time to clot, increased platelet function approaching term, and increased resistance to fibrinolysis. This discrepancy between this patient's trends and the expected gestational physiology indicates that fetal reduction might initiate a change in fibrinolysis throughout pregnancy.

These observations are notable when contextualized within the prior literature. Studies on healthy term pregnancies have consistently reported higher clot strength (MA) values compared with those in non-pregnant individuals, reflecting increased platelet function as gestation advances. Our data reflect values similar to those of Davies et al. (11), who reported a mean MA of 73 mm in healthy term pregnancies, which is considered hypercoagulable compared to the normal range supplied by the manufacturer. Our data are also similar to those of Gui et al. (12), who observed a mean MA of 68.7 mm in pregnant individuals and a mean MA of 62.7 mm in non-pregnant individuals. The observed reduction in clot strength following FR suggests that FR may play a role in reducing the development of a hypercoagulable state that occurs with multiple gestations.

Furthermore, in comparing our fibrinolysis data with those of Gui et al. (12), it was observed that patient 611 at term exhibited an LY30 value similar to that of healthy controls rather than pregnant individuals. Our findings show that differences in fibrinolysis in FR compared to controls are most dramatic at the end of pregnancy. This suggests that fetal reduction could induce changes in fibrinolysis that occur throughout pregnancy. The broad potential clinical implications of these findings include better risk stratification in higher-order pregnancies, more informed decision-making regarding pharmacologic thromboprophylaxis, and ultimately, a reduction in maternal morbidity.

This report has several strengths, including detailed longitudinal TEG data, consistent sampling within a prospective protocol, and the availability of an internal control cohort conceived using the same reproductive method. Nonetheless, the findings must be interpreted within the inherent limitations of a single case report, as conclusions drawn from individual cases are necessarily of limited generalizability and should be viewed within this context. Given the nature of the case study data, our limited statistical analysis is restricted to reporting descriptive statistics of duplicate measures for the case's TEG parameters and a box and whisker plot presenting the variation of the IUI control group (Figure 2). While our TEG data assessed the overall hemostatic potential, we did not identify the specific coagulation or fibrinolysis factors underlying the observed changes. Therefore, our findings are hypothesis-generating, and the descriptive nature of our analysis precludes causal or mechanistic inferences. The temporal alignment of TEG sampling between the cases and controls was not precise and was not replicated in the second trimester, and unmeasured clinical factors may have contributed to the observed differences. In addition, without TEG data from unreduced triplet pregnancies, it remains unknown whether the patient's initial hemostatic trajectory prior to reduction mirrored typical patterns for higher-order gestations.

Despite these limitations, this case highlights an important and previously unreported area of physiological research. As fetal reduction remains an established option for managing higher-order multiple pregnancies, understanding how the procedure influences coagulation may have clinical relevance, particularly in the context of pregnancy-related thrombotic risk. Larger prospective studies are required to determine whether the patterns observed in this study are consistent across individuals undergoing FR and to explore the underlying mechanisms.

## Data Availability

Data will not be made publicly available. Research inquiries made to the senior author will be considered on a case by case basis.
